# Vitamin D Level and Activities of Daily Living in Octogenarians: Cross-Sectional Study

**DOI:** 10.3389/fendo.2018.00326

**Published:** 2018-06-13

**Authors:** Vidmantas Alekna, Justina Kilaite, Asta Mastaviciute, Marija Tamulaitiene

**Affiliations:** Faculty of Medicine, Vilnius University, Vilnius, Lithuania

**Keywords:** age, octogenarians, total 25 hydroxyvitamin D, activities of daily living, instrumental activities of daily living

## Abstract

**Introduction:**

Despite the growing number of octogenarians, little is known about their vitamin D status and activities of daily living (ADL) relations.

**Objective:**

The aim of this study was to investigate peculiarities of vitamin D and ADL and to assess their relations in octogenarians.

**Methods:**

A cross-sectional study was performed at the National Osteoporosis Centre located in Vilnius, Lithuania. Community-dwelling ambulatory persons aged ≥80 years were included. Current users of vitamin D supplements were excluded. Total 25 hydroxyvitamin D concentration in serum was measured with Cobas E411. Functional status was assessed by Katz ADL and the Lawton Instrumental Activities of Daily Living (IADL) scales. Subjects were divided into three groups according to age and into two groups according to vitamin D level. One-way analysis of variance with *post hoc* test was used to determine between-group comparisons. Associations between vitamin D and ADL score, and IADL score were assessed using Spearman’s correlation.

**Results:**

The study was performed on 153 octogenarians: 81 (52.9%) women and 72 (47.1%) men. The average age of subjects was 83.9 ± 3.2 years. Mean total 25 hydroxyvitamin D concentration was 11.2 ± 7.0 ng/ml; 137 (89.5%) persons had vitamin D deficiency, 12 (7.8%) had insufficiency, and only 4 (2.6%) persons were vitamin D sufficient. Positive weak correlation between total 25 hydroxyvitamin D and ADL score (*r* = 0.2, *p* = 0.01) and very weak correlation between total 25 hydroxyvitamin D and IADL score (*r* = 0.19, *p* = 0.02) were found. Total 25 hydroxyvitamin D level was correlated with ADL score in women (*r* = 0.23, *p* = 0.04). In the 80–84 years group ADL score correlated with total 25 hydroxyvitamin D level (*r* = 0.23, *p* = 0.02).

**Conclusion:**

The majority of investigated octogenarians had vitamin D deficiency. The level of vitamin D was associated with the ADL score. There was no association between the vitamin D level and the IADL score, although a weak correlation was found between vitamin D level and category of food preparation.

## Introduction

There were 137 million persons aged 80 years or above (octogenarians) living in the world in 2017, and this number is projected to increase more than threefold in 2050, to 425 million (1). In Lithuania, there were 146,319 octogenarians (5.19% of total population) and 33,751 (1.2%) of these were living in the capital city Vilnius living at the end of 2017 (2). It is estimated that 1 billion people have vitamin D deficiency or insufficiency worldwide, and this is particularly prevalent among elderly people (3). Octogenarians are at risk of total 25 hydroxyvitamin D deficiency for several reasons: a tendency to avoid the sun; decreased skin capacity to produce vitamin D; and diminished intestinal absorption and/or decreased vitamin D dietary intake (4). There is growing evidence that total 25 hydroxyvitamin D deficiency is associated with muscle weakness, increased risk of falling, impaired functional status, lower cognitive performance score, frailty, and poorer quality of life in elderly people (5). The association between total 25 hydroxyvitamin D deficiency and mortality is inconclusive, as some studies report a positive relationship while others do not (6, 7).

Aging is associated with decreased muscular strength and physical function, these changes lead to decreased mobility and independence, such as difficulties in walking and transferring from a bed or chair (8). Activities of daily living (ADL) include the fundamental skills typically needed to manage basic physical needs, comprised of various functional skills in different areas (9).

Despite the growing number of octogenarians, little is known about their vitamin D status and ADL relations. There are few studies performed in the oldest-old group regarding information about the total 25 hydroxyvitamin D status and functional independence relationship (10–14). In three of these studies a positive association between vitamin D level and ADL was found. Houston and colleagues (10) and Nakamura and colleagues (11) revealed that vitamin D deficiency was a predictor of low ADL scores in community-dwelling adults of advanced age. Kotlarczyk and colleagues showed that women in long-term care facilities, who had low levels of vitamin D, had the greatest functional decline (12). In another two studies no association between vitamin D and ADL were found. Navarro-Martínez and colleagues did not find an association between the vitamin D level and ADLs in frail octogenarian women (13). While Formiga and colleagues did not show any association between vitamin D status and IADLs, a positive relationship between vitamin D level and ADL score was found (14).

The aim of this study was to investigate the peculiarities of vitamin D and ADL and to assess their relations in octogenarians.

## Materials and Methods

This cross-sectional study was conducted at the National Osteoporosis Centre, Vilnius, Lithuania from January 2017 to February 2018. Non-probability convenience sampling method was used for the study population. Subjects were enrolled from outpatient clinics in Vilnius city. Inclusion criteria were: age 80 years and above; community-dwelling ambulatory women and men. The exclusion criteria were: moderate cognitive impairment (mini-mental state examination score < 21); acute illness; chronic diseases in terminal stages; and current use of vitamin D supplements. Subjects were divided into three age groups: aged 80–84 years, 85–89 years, and above 90 years. The height was measured barefoot with a stadiometer to the nearest 0.1 cm. Body weight was measured barefoot and in light indoor clothes with an electronical medical scale to the nearest 0.05 kg. Body mass index (BMI) was calculated as weight in kilograms divided by the height in meter squared (kg/m^2^).

Blood samples were collected from 8:00 a.m. till 11:00 a.m., after fasting for at least 12 h. Serum total 25 hydroxyvitamin D (vitamin D) was measured by automated immunoassay (Cobas E411, Roche Diagnostic). Analysis was carried out by fully automated electrochemical luminescence immunoassay method using the original reagents, and in accordance with the manufacturer’s instructions, regular calibration, and quality control applied on a daily basis. The total coefficient of variation was 5.9%.

The Endocrine Society Guidelines were used to interpret the total 25 hydroxyvitamin D level in serum: 25(OH)D concentration of 20 ng/ml (50 nmol/l) or below was defined as vitamin D deficiency; vitamin D insufficiency was determined as 25(OH)D concentration of 21–29 ng/ml (52.5–72.5 nmol/l); and concentration of vitamin D equal or above 30 ng/ml (75 nmol/l) was assessed as sufficient (15). In this study, we pooled sufficiency and insufficiency groups. Seasons were defined as winter (December–February), spring (March–May), summer (June–August), and autumn (September–November).

The functional status was evaluated using Katz ADL scale: bathing, dressing, toileting, transferring, continence, and feeding (16). Lawton Instrumental Activities of Daily Living categories were as follows: ability to use telephone; shopping; food preparation; housekeeping; laundry; mode of transportation; responsibility for own medications; and ability to handle finances (17).

The study protocol has been approved by Vilnius Regional Biomedical Research Ethics Committee. All subjects gave their written informed consent prior to enrollment.

Statistical analysis was performed using IBM SPSS Statistics Windows software version 18 (IBM, New York). All data were expressed as mean, SD, or frequencies (number, percentage), as appropriate. Distribution of continuous variables was assessed by the Shapiro–Wilk test. Mean differences of interval variables were compared using Student’s *t*-test. The one-way analysis of variance (ANOVA) was used to determine whether there were any significant differences between the means of three or more independent groups. Associations between vitamin D levels and ADL, IADL scores were assessed using Spearman correlation analysis. The level of significance (*p*-value) of <0.05 was considered as statistically significant.

## Results

In total, 153 persons participated in this study: 81 women (52.9%) and 72 men (47.1%). Age ranged from 80.0 to 95.6 years: 80.0–92.5 years for women and 80.1–95.6 years for men. Basic descriptive characteristics of study population are shown in Table 1. Upon further investigation, none of the IADL categories could be pointed out as cause for difference in IADL scores between women and men.

**Table 1:** Basic descriptive characteristics of study population (mean ± SD).

Characteristic	All subjects (*n* = 153)	Women (*n* = 81)	Men (*n* = 72)	*p*
Age (years)	83.89 ± 3.18	83.49 ± 2.61	83.94 ± 3.43	0.36
Height (cm)	162.61 ± 9.48	156.29 ± 7.52	169.71 ± 5.65	<0.001
Weight (kg)	72.04 ± 11.81	68.41 ± 12.37	76.13 ± 9.68	<0.001
BMI (kg/m^2^)	27.29 ± 4.38	28.07 ± 5.13	26.42 ± 3.17	0.02
Total 25 hydroxyvitamin D (ng/ml)	11.15 ± 7.01	10.61 ± 6.85	11.76 ± 7.19	0.46
– Deficiency	9.41 ± 4.41	9.27 ± 4.5	9.59 ± 4.32	0.36
– Insufficiency	22.19 ± 2.74	21.55 ± 1.39	22.51 ± 3.26	0.23
– Sufficiency	32.42 ± 1.67	33.59 ± 1.52	31.25 ± 0.81	0.29
ADL (score)	4.5 ± 1.07	4.57 ± 1.11	4.43 ± 1.03	0.43
IADL (score)	5.72 ± 2.42	6.12 ± 2.12	5.26 ± 2.67	0.03

*p-Value was calculated using Student’s t-test when comparing women and men*.

*BMI, body mass index; ADL, activities of daily living; IADL, instrumental activities of daily living*.

No association was found between seasons of blood sampling (winter, spring, summer, and autumn) and vitamin D level (*p* = 0.65). In spring, total 25 hydroxyvitamin D was collected from 55 (35.9%) subjects, and their mean total 25 hydroxyvitamin D level was 11.64 ± 8.02 ng/ml. In summer, 24 (15.7%) subjects’ blood was sampled and mean total 25 hydroxyvitamin D level was 10.68 ± 6.23 ng/ml. The total 25 hydroxyvitamin D level of 44 (28.8%) subjects’ was collected in autumn and mean total 25 hydroxyvitamin D level was 10.4 ± 6.04 ng/ml. In winter, 30 (19.6%) subjects’ blood was collected and their mean total 25 hydroxyvitamin D level was 11.73 ± 7.14 ng/ml. No differences between total 25 hydroxyvitamin D levels were found in women and men. Results of the analysis of the study population in the different age groups are shown in Table 2.

**Table 2:** Characteristics of study population in age groups (mean ± SD).

Characteristic	80–84 years (*n* = 107)	85–89 years (*n* = 38)	90+ years (*n* = 8)	Analysis of variance *p*
Age (years)	82.71 ± 1.38	87.03 ± 1.39	92.21 ± 2.17	<0.001
BMI (kg/m^2^)	28.07 ± 4.29	25.63 ± 4.19	24.78 ± 3.72	<0.001
Total 25 hydroxyvitamin D (ng/ml)	11.23 ± 6.65	10.45 ± 8.11	13.37 ± 6.42	0.55
ADL (score)	4.57 ± 0.98	4.47 ± 1.2	3.75 ± 1.48	0.11
IADL (score)	5.86 ± 2.37	5.58 ± 2.43	4.5 ± 3.02	0.29

*BMI, body mass index; ADL, activities of daily living; IADL, instrumental activities of daily living*.

Age groups did not differ in total 25 hydroxyvitamin D levels, ADL, and IADL scores. The mean total 25 hydroxyvitamin D level in all age groups was deficient. BMI was higher in the 80–84 years age group compared to the 85–89 years age group (*p* = 0.01). No differences were found between genders.

It was found that in the age group from 80 to 84 years, 94 (87.9%) people were vitamin D deficient, 11 (10.3%) were in the insufficiency group and only 2 (1.87%) octogenarians had sufficient vitamin D levels. In the age group ranging from 85 to 89 years, 36 (94.74%) subjects had deficient levels of vitamin D, none were in the insufficiency category and 2 (5.26%) had normal levels of vitamin D. In the oldest group (90+ years), 7 subjects (87.5%) had vitamin D deficiency, 1 (12.5%) subject had insufficient levels of vitamin D and none of them had sufficient levels of vitamin D.

Of all the subjects, 137 (89.5%) octogenarians had vitamin D deficiency, in 12 persons (7.8%) insufficiency was found, and only 4 (2.6%) subjects were vitamin D sufficient. Characteristics of the study population in vitamin D status groups are shown in Table 3.

**Table 3:** Characteristics of study population in vitamin D status groups (mean ± SD).

Characteristic	Sufficient/insufficient (*n* = 16)	Deficient (*n* = 137)	*p*
Age (years)	83.53 ± 2.78	83.94 ± 3.23	0.59
BMI (kg/m^2^)	26.21 ± 3.83	27.42 ± 4.44	0.25
Total 25 hydroxyvitamin D (ng/ml)	24.74 ± 5.2	9.41 ± 4.4	<0.001
ADL (score)	4.69 ± 0.94	4.44 ± 1.1	0.02
IADL (score)	6.88 ± 1.2	5.55 ± 2.45	<0.001

*BMI, body mass index; ADL, activities of daily living; IADL, instrumental activities of daily living*.

No differences were found between women and men in the vitamin D status groups. Furthermore, there was no difference between age and BMI. Further analysis showed that subjects with vitamin D deficiency scored lower in ADL than subjects with vitamin D insufficiency (*p* = 0.02) or sufficiency (*p* < 0.001). The difference in the ADL score between insufficiency and sufficiency groups was not significant. The IADL score in the vitamin D deficiency group was significantly lower in the sufficiency group (*p* < 0.001), although there was no difference with the insufficiency group. The vitamin D insufficiency and sufficiency groups did not differ in IADL score.

**Figure 1:**
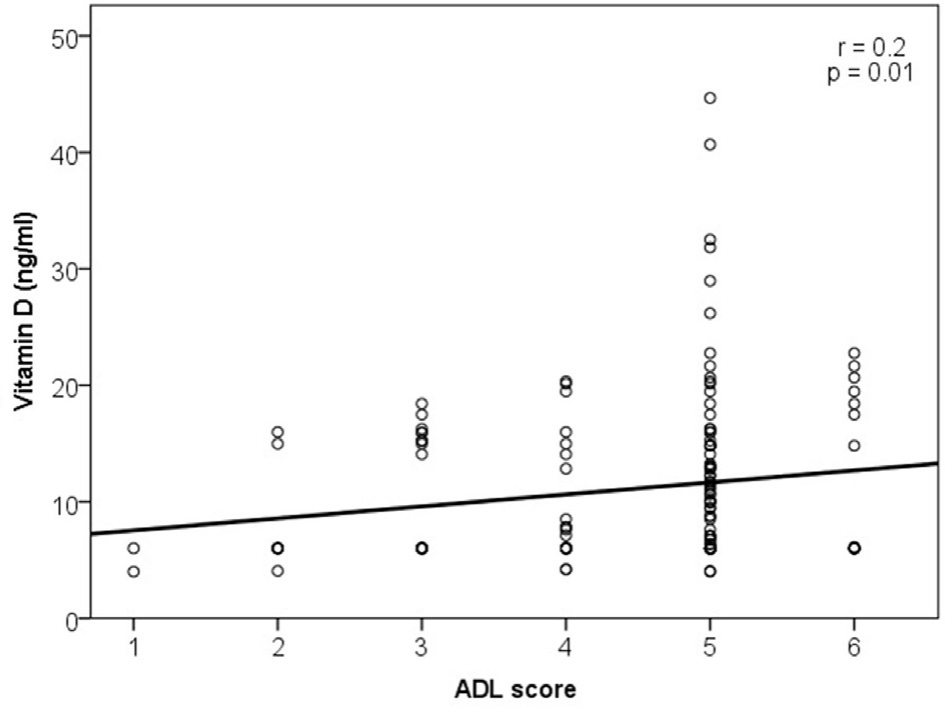
Association of vitamin D level with activities of daily living (ADL).

**Figure 2:**
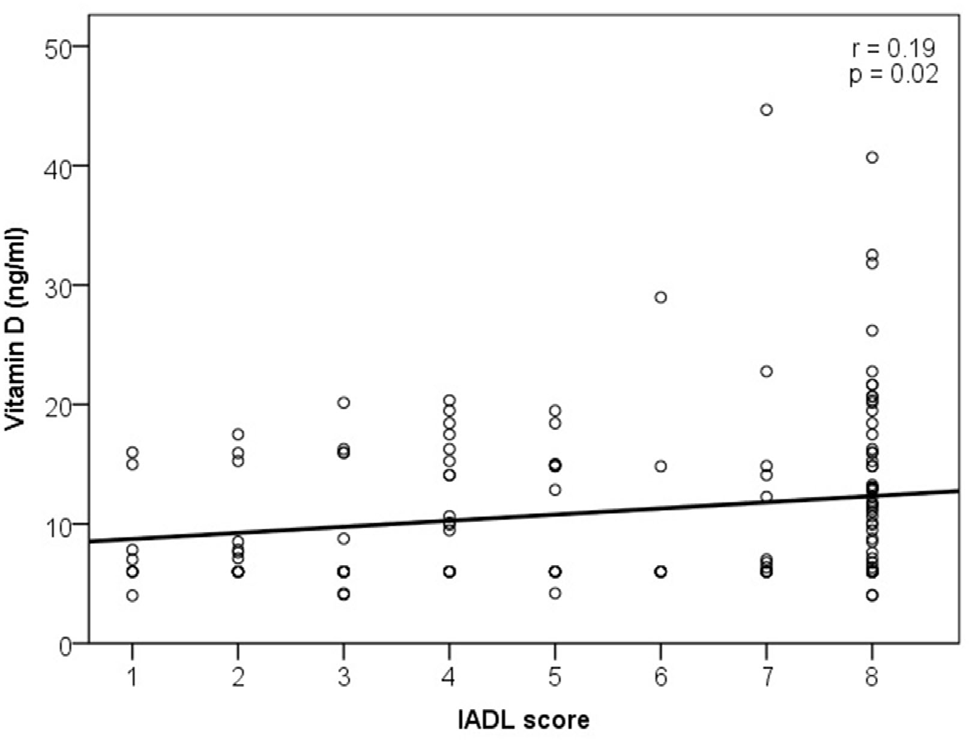
Association of vitamin D level and instrumental activities of daily living (IADL).

Vitamin D correlations with ADL and IADL are shown in Figures 1 and 2, respectively.

A weak correlation was found between the vitamin D and ADL score (*r* = 0.2, *p* = 0.01) and very weak between the vitamin D and IADL score (*r* = 0.19, *p* = 0.02). There was no association between the serum vitamin D level and any single ADL category (bathing, dressing, toileting, transferring, continence, and feeding). Statistically significant correlations were found in two of eight IADL categories: weak correlation of vitamin D with food preparation (*r* = 0.2, *p* = 0.02) and very weak with the ability to use telephone (*r* = 0.19, *p* = 0.02).

Further analysis revealed a weak correlation between the vitamin D level and ADL score in women (*r* = 0.23, *p* = 0.04), and no correlations were found in men. When vitamin D level associations with ADL and IADL scores were analyzed in different age groups, correlation was found only for the ADL score in the 80–84 years group (*r* = 0.23, *p* = 0.02). No correlation between vitamin D level and ADL or IADL scores was found in different vitamin D status groups.

## Discussion

The results of our study show a high prevalence of vitamin D deficiency in community-dwelling ambulatory subjects aged 80 years and older—it was found in more than 89% of study subjects.

Data on the vitamin D status of oldest-old adults are scarce: only a few studies were aimed to investigate the vitamin D status in octogenarians (10, 13, 17–20). Some other investigators had also found quite a high amount of vitamin D deficiency in 52.5–80.9% of oldest-old subjects investigated, depending on region (4, 6). Usually vitamin D deficiency is investigated in elderly with specific conditions.

Bruyère and colleagues (19) have reported a high prevalence of vitamin D inadequacy in 1984 European women aged over 80 years with osteoporosis and osteopenia. Results of this study showed that the average level of total 25 hydroxyvitamin D was 21.4 ng/ml, and different levels have been shown for different European countries. Our data show that the mean vitamin D level in women (10.61 ng/ml) was lower than the lowest level found in Belgian women (18.3 ng/ml). The difference of results could be explained by different study populations: only women with osteopenia and osteoporosis were investigated in the study conducted by Bruyère and colleagues (19), while we have included subjects regardless of their illness. Moreover, we did not include persons taking vitamin D supplementation.

Navarro-Martínez and colleagues (13) reported the significantly reduced vitamin D concentration in frail institutionalized women. Even in frail women, authors have found higher levels of vitamin D (28 ng/ml) as compared to our results. Bruyère and colleagues (19) have also found higher concentrations in Spain women—32.7 ng/ml. These differences might be explained by different geographical area: study was performed in Spain which is located at lower latitude than Lithuania.

In oldest-old Americans vitamin D deficiency was lower compared to their European counterparts and was found in 21.5% of subjects (18). A higher prevalence of vitamin D deficiency is seen in women rather than in men (75.6 vs. 24.4%) (6). Also, some studies found that an age-associated fall in serum total 25 hydroxyvitamin D starts earlier in women than in men (10, 21). In our study no difference in vitamin D levels were found between octogenarian men and women.

Results of this study show a weak correlation between total 25 hydroxyvitamin D levels and ADL in women. According to the results of Navarro-Martínez and colleagues (13), no correlation between vitamin D levels and frailty syndrome was found in institutionalized octogenarian women. Nakamura and colleagues (11) reported that low serum vitamin D levels were associated with low ADL levels, but ADL was assessed in a younger population and using a different scale—the Barthel index. Kotlarczyk and colleagues (12) investigated the association of vitamin D deficiency with functional changes assessed by ADLs and IADLs. The authors concluded that women with vitamin D deficiency had a greater decline in physical function. However, the study population was very different from our subjects in number and social status (residing in long-term care facilities). Cardiovascular Health Study All Stars (10) revealed that older adults with deficient total 25 hydroxyvitamin D levels were approximately 50% more likely to report ADL disability. However, most of the studies were cross-sectional in design and it is difficult to assess whether low vitamin D levels preceded the onset of reported functional skills or whether individuals had low total 25 hydroxyvitamin D levels because their functional status was poorer and, because of limited time outdoors, had less exposure to the sun and less endogenous vitamin D synthesis. No association between serum vitamin D and any single ADL or IADL categories was found, and further research with a large study population is needed.

The strength of our study is that we have investigated female and male octogenarians without vitamin D supplementation.

Our study also has some limitations. The results of this study could not represent the whole Lithuanian population. We have not assessed the physical activity and muscle strength of study subjects, or nutritional status. This would help to make a more comprehensive assessment of octogenarians.

In conclusion, this study reports a high prevalence of vitamin D deficiency in the studied octogenarians. Our findings suggest a weak correlation between vitamin D and activity of daily living. Weak correlation was found between vitamin D levels and instrumental activities of daily living category of food preparation. Further studies are needed to address the issue.

## Ethics Statement

Study protocol has been approved by Vilnius Regional Biomedical Research Ethics Committee. All participants gave their written informed consent prior to enrolment.

## Author Contributions

VA, AM, and MT were involved in the design of the study. VA, JK, and AM were involved in the management of the study. JK and AM performed the statistical analysis. VA, JK, AM, and MT drafted the manuscript and contributed in the final manuscript. All authors read and approved the final manuscript.

## Conflict of Interest Statement

The authors declare that the research was conducted in the absence of any commercial or financial relationships that could be construed as a potential conflict of interest.
